# A phase 1 trial of recombinant human IL-21 in combination with cetuximab in patients with metastatic colorectal cancer

**DOI:** 10.1038/bjc.2011.599

**Published:** 2012-02-07

**Authors:** N Steele, A Anthony, M Saunders, B Esmarck, E Ehrnrooth, P E G Kristjansen, A Nihlén, L T Hansen, J Cassidy

**Affiliations:** 1 CRUK Clinical Trials Unit, Beatson West of Scotland Cancer Centre, 1053 Great Western Road, Glasgow G12 0YN, UK; 2 Medical Oncology CRUK Clinical Cancer Centre, Leeds, UK; 3 Christie Hospital, Manchester, UK; 4 Novo Nordisk, A/S, Copenhagen, Denmark

**Keywords:** immunotherapy, colorectal cancer, rIL-21, cetuximab

## Abstract

**Background:**

Pre-clinical data indicate enhanced anti-tumour activity when combining recombinant human interleukin-21 (rIL-21), a class 1 cytokine, with cetuximab, a monoclonal antibody, targeting the epidermal growth factor receptor. This phase 1 trial assessed the safety and tolerability of escalating doses of rIL-21 in combination with cetuximab in chemo-naïve patients with stage IV colorectal cancer.

**Patients and methods:**

Sequential cohorts of PS 0–1, asymptomatic patients, were treated weekly with cetuximab 250 mg m^−2^ intravenously (i.v.) plus escalating i.v. doses of rIL-21 following an initial loading dose of cetuximab 400 mg m^−2^. Initial treatment period was 8 weeks, with extension permitted in patients without disease progression.

**Results:**

In all, 15 patients were included in this study. Adverse events related to rIL-21 or rIL-21 plus cetuximab included lethargy, nausea/vomiting, stomatitis, lymphopenia and pyrexia and were mainly ⩽ grade 2. One dose limiting toxicity occurred (grade 3 diarrhoea). Maximum tolerated dose was not determined because of the premature study closure. Maximum administered dose was 100 *μ*g kg^−1^ rIL-21 weekly. In all, 60% of the patients had stable disease. Immune activation was confirmed by various T- and NK-cell activation biomarkers, including dose-dependent increases in serum sCD25.

**Conclusion:**

rIL-21 weekly combined with cetuximab is well tolerated at doses up to 100 *μ*g kg^−1^ and results in activation of immune response biomarkers.

Colorectal cancer (CRC) is the third commonest cancer worldwide, and is the fourth commonest cause of cancer death. As with many cancers, surgical resection is the primary treatment for localised disease, leading to cure rates of around 50%. Unfortunately, at least 20% of patients have metastatic disease at the time of presentation and a further 50% of those presenting with localised disease will relapse despite surgery and adjuvant chemotherapy +/or radiotherapy. Although a minority of patients will have isolated, resectable metastases, for the majority cure is not a possibility and treatment is palliative. Systemic therapy of metastatic CRC has been evolving rapidly in recent years. A widening range of treatment options has led to improvements in survival, with median survival now approaching 2 years in patients who are fit to receive multiple lines of therapy. Systemic therapy for metastatic CRC is currently based on three chemotherapy agents (5-fluorouracil, oxaliplatin and irinotecan) and two monoclonal antibodies (mAbs; bevacizumab and cetuximab).

In some patients, metastases from CRC are diagnosed on the basis of a rising tumour marker, carcinoembryonic antigen (CEA), or abnormal imaging despite remaining largely asymptomatic. The optimum timing of systemic chemotherapy in such patients is unclear. Two studies published in the 1990s were designed to address this issue but results were conflicting and do not reflect existing treatment strategies (Ackland *et al*, 2005). It is generally accepted that in balancing clinical data, patient preference and potential treatment toxicity, it is justifiable to postpone chemotherapy until symptomatic progression in some patients. There exists, therefore, a ‘window of opportunity’ in which novel therapeutic strategies may be explored. In this study we have used this opportunity to test an approach known as ‘first-line experimental therapy’.

One such therapeutic strategy is immunotherapy, which may have potential in patients who are not heavily pre-treated with chemotherapy and hence have a relatively competent immune system. In this phase 1 trial we have investigated the combination of recombinant human interleukin-21 (rIL-21) and cetuximab in asymptomatic patients with metastatic CRC who did not require immediate chemotherapy.

IL-21 is a class 1 cytokine, which is produced by activated CD4+ T cells *in vivo*. IL-21 stimulation of T cells, B cells and NK cells leads to enhanced proliferation and mature effector function. The drug substance rIL-21 is a human recombinant interleukin, which has been investigated in pre-clinical and clinical studies as a single agent. In a phase 1 trial in patients with metastatic melanoma, rIL-21 was generally well tolerated with dose limiting toxicities (DLTs) including raised liver transaminases, fatigue, neutropenia and thrombocytopenia. The maximum tolerated dose (MTD) was 30 *μ*g kg^−1^ when given either thrice weekly or for days 1–5 of a 14-day cycle (Davis *et al*, 2007).

Cetuximab is a recombinant chimeric immunoglobulin G1 mAb. The binding of cetuximab to the epidermal growth factor receptor (EGFR) results in the inhibition of cell growth, induction of apoptosis and decreased matrix metalloproteinase and vascular endothelial growth factor production. Furthermore, cetuximab can trigger tumour-directed cytotoxic immune response of Fc receptor expressing immune effector cells (e.g. NK cells), which leads to antibody-dependent cell-mediated cytotoxicity (ADCC) of tumour tissue. Cetuximab, administered intravenously (i.v.) on a weekly basis, improves tumour response rate and progression-free survival when combined with standard chemotherapy for metastatic CRC (Bokemeyer *et al*, 2009; Van Cutsem *et al*, 2009). Cetuximab has also been demonstrated to have activity as a single agent in patients who have previously been treated with a fluoropyrimidine, irinotecan and oxaliplatin (Jonker *et al*, 2007).

ADCC may be a significant factor in the activity of cetuximab in CRC. Pre-clinical *in vitro* data have demonstrated that cetuximab-mediated NK-cell activity can be significantly enhanced in the presence of IL-21 (Roda *et al*, 2007; Watanabe *et al*, 2010). The combination of rIL-21 and cetuximab would *in vivo* potentially enhance such ADCC mechanism and augment the immune response towards malignant cells (Figure 1). The primary objective of this phase 1 trial was to determine the safety, toxicity and MTD of rIL-21 in combination with cetuximab in patients with metastatic CRC. Secondary objectives were to determine the pharmacokinetic (PK) and pharmacodynamic characteristics of rIL-21 in combination with cetuximab, the potential for immune activation, as measured by a series of markers of immune function, and to evaluate the potential efficacy of this regimen.

## Patients and methods

### Eligibility

This study was approved by the research ethics committees at all three participating institutions. Eligible patients were those with stage 4 histologically-confirmed adenocarcinoma of the colon or rectum aged 18 years or older with ECOG performance status ⩽1 and an estimated life expectancy of >3 months. Patients included were asymptomatic and those in whom a delay in starting chemotherapy was ethically and medically justifiable. Written informed consent was obtained from all study participants. Adequate bone marrow, hepatic and renal function as defined for trial entry was as follows: white blood cell ⩾2.5 × 10^9^ l^−1^, absolute neutrophil count ⩾1.5 × 10^9^ l^−1^, platelet count ⩾100 × 10^9^ l^−1^, haemoglobin ⩾6.2 mmol l^−1^, lymphocytes 0.8 × 10^9^ l^−1^, serum creatinine ⩽177 *μ*mol l^−1^, bilirubin ⩽2.5 × upper limit of normal (ULN), AST/SGOT ⩽2.5 × ULN unless liver metastases in which case ⩽5 × ULN and LDH ⩽2 × ULN. Tumour K-ras status was not tested.

Patients were excluded from the study if they had potentially resectable metastases, a requirement for immediate chemotherapy or signs of CNS metastases. Patients who had had a prior chemotherapy for stage IV CRC were excluded and in those who had received adjuvant chemotherapy, 6 months had to have elapsed since the end of treatment. Prior radiotherapy for metastases was permitted provided at least 4 weeks had elapsed since treatment of bony metastases or 8 weeks in the case of visceral metastases. Prior treatment with cetuximab or any other targeted EGFR blocking agent was not permitted. Patients with autoimmune disease (excluding vitiligo and treated pernicious anaemia) or documented positive serologic testing for hepatitis B or HIV were ineligible for the study. Other exclusion criteria were cardiac failure (NYHA III or IV), unstable angina or myocardial infarction in the last 12 months, clinically uncontrolled infection, concurrent corticosteroid therapy, pregnancy/breastfeeding or any other significant systemic disease, which could compromise patient safety or interfere with study procedures.

### Study design

**Figure 2:**
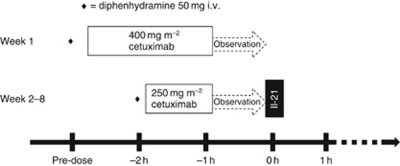
Dosing schedule for cetuximab and rIL-21.

This was a phase 1, multi-centre, open label study designed to evaluate the safety, PKs and pharmacodynamics of escalating doses of rIL-21 in combination with cetuximab in patients with stage IV CRC, who had not received chemotherapy for metastatic disease. A weekly dosing schedule for rIL-21 was chosen based on standard scheduling of cetuximab. Sequential cohorts of three patients were entered into one of the escalating rIL-21 dose steps, which were planned to be 3 *μ*g kg^−1^, 10 *μ*g kg^−1^, 30 *μ*g kg^−1^, 100 *μ*g kg^−1^, 200 *μ*g kg^−1^ and 300 *μ*g kg^−1^ (Figure 2). Escalation from one dose level of rIL-21 was permitted once all subjects in the cohort had been monitored for toxicity for a minimum of 4 weeks. No intra-patient dose escalation was permitted.

After pre-medication with an H1 antagonist (systemic steroid pre-medication was prohibited), subjects were initiated with a loading dose of cetuximab 400 mg m^−2^ as an i.v. infusion over 120 min one week in advance of rIL-21 treatment, thereby allowing discrimination between cetuximab-related infusion reactions and IL-21-related adverse events (AEs). Subsequent weekly doses of cetuximab 250 mg m^−2^ were given as 60 min i.v. infusions and patients observed for 1 h before dosing with rIL-21 given i.v. as a bolus. Patients were observed for at least 1 h after dosing with rIL-21.

Weekly doses of cetuximab were lowered if a subject experienced ⩾grade 3 skin toxicity between treatments, provided this had resolved to ⩽grade 2 before scheduled dosing one week later. Where this occurred following the loading dose of cetuximab, no modification was made to the subsequent weekly dose unless further episodes of grade ⩾3 skin toxicity occurred, whereby dose reductions to 200 mg m^−2^ then 150 mg m^−2^ could be made. If severe skin toxicity occurred for a fourth time or did not resolve to ⩽grade 2 between treatments, the patients were withdrawn from the trial.

### DLT and definition of MTD

Adverse events were graded and reported according to the National Cancer Institute Common Terminology for adverse events (CTCAE) version 3.0. In general, grade 3 AEs were considered to be dose limiting with the exception of the following recognised effects of rIL-21: grade 3 fever, grade 3 asymptomatic hyperglycemia, grade 3 lymphopenia and grade 3 neutropenia. The MTD was defined at the outset of the trial as the highest dose level administered where ⩽1 out of 6 subjects has a DLT.

### Patient assessments and response criteria

The primary objective of the trial was assessment of safety and tolerability. In patients with measurable disease, the effect on the tumour was assessed radiologically according to RECIST criteria. In all patients, disease assessment was carried out at baseline and after 8 weeks of treatment. Weekly clinical assessment and CEA monitoring was carried out to detect early indicators of disease progression in-between formal imaging visits. In case of tumour progression, patients were taken off the study immediately and appropriate standard treatment was instituted. In the absence of tumour progression after 8 weeks on trial, an additional treatment period of 8 weeks was permitted, with subsequent extensions of treatment following a second tumour evaluation being at the discretion of the investigator. If, after 16 weeks of study treatment, there was evidence of ongoing clinical benefit, continued treatment with rIL-21 and cetuximab could be offered on a named patient basis.

### Drug preparation

#### IL-21

rIL-21 was provided by Novo Nordisk A/S (Copenhagen, Denmark) in vials containing 0.8 ml of 10 mg ml^−1^ solution. rIL-21 vials were stored in a freezer at −20 °C until required for use. At dose levels up to 30 *μ*g kg^−1^, the product was diluted to 0.1 or 1 mg ml^−1^ using sterile saline for injection (sodium chloride 0.9%, w/v). At higher doses, no dilution was required.

#### Cetuximab

Erbitux Merck (Darmstadt, Germany) (cetuximab) was supplied in single-use, 50 ml vials containing 100 mg of cetuximab at a concentration of 2 mg ml^−1^ in a preservative free solution. Cetuximab vials were stored under refrigeration (2–8 °C).

### Pharmacokinetic studies

Pharmacokinetic samples for rIL-21 and cetuximab were taken at visits 3 and 8 at the following time-points: pre-dose of cetuximab, at the end of cetuximab infusion (−1 h) and at 0, 5, 15 and 30 min and 1, 2, 4, 8, 12 and 24 h post rIL-21 bolus. Further cetuximab PK samples were taken at pre-dose at visits 4 and 9. Enzyme-linked immunosorbent assay was used to analyse serum samples of rIL-21 and cetuximab. Pharmacokinetic parameters were determined using non-compartmental methods and a population PK modelling approach.

### Pharmacodynamic biomarker studies

All pharmacodynamic biomarker studies were exploratory in nature. Cytotoxicity towards NK-sensitive K562 target cells, activation markers on NK cells and monocytes, as well as soluble IL-2 receptor-alpha (sCD25) were measured at Esoterix Clinical Trial Services, Belgium.

Blood samples for all biomarker analyses were collected pre-dose in the main trial at visit 2, 3 and 4 and for sCD25 also at all other dosing visits. Additional biomarker samples for NK cytotoxicity, flow cytometry of NK cells and monocytes, and serum sCD25 were also taken 24 h after rIL–21 dosing on visit 3. During extension treatment, blood samples were collected pre-dose for sCD25 at weeks 9, 11, 13, 15 and the final follow-up visit.

Serum samples for anti-rIL-21 antibody analysis were analysed by Covance Laboratories Ltd (Harrogate, UK). Serum antibodies specific for rIL–21 were measured by enzyme-linked immunosorbent assay before dosing in weeks 2, 4, 6 and 8 in the main trial and at week 16 in the extension treatment period. Positive antibody sera were to be subsequently analysed for rIL-21 neutralising antibody.

## Results

### Patient characteristics

In all, 15 patients (13 male and 2 female) with stage IV CRC were entered into the study between February 2007 and September 2008. Median age was 66 years (range 44–83). The ECOG performance status was zero in 10 patients, the remainder having a performance status of one. All subjects had prior surgery for CRC. In eight patients, the primary tumour was in the colon and seven patients had a rectal as primary. In all, 8 patients had prior radiotherapy and 13 had prior chemotherapy in the adjuvant setting. Median time from first diagnosis of CRC was 1.4 years (range 0.7–6.7 years). The majority of patients (13) had stage I–III disease at the time of initial diagnosis. Two patients had presented initially with stage IV disease and had their diagnosis made from biopsy of a metastatic lesion (one in the liver and one in the lung). Subjects received a median of nine doses of rIL-21 (range 4–15 doses).

### Protocol violations

There were two instances in which the protocol was violated. One subject was included in the trial in error despite not being eligible, as he had received adjuvant chemotherapy within the last 6 months. It was believed that the chemotherapy did not have major impact on the tumour response and the subject completed both the week 8 and week 16 tumour assessment. The tumour response data were also included in the analysis of the tumour responses, the best overall response being stable disease. In addition to this, one patient was treated with i.v. hydrocortisone for safety reasons following a suspected reaction to cetuximab. Continuation in the study was evaluated to be in the interest of the subject and did not pose any safety concerns.

### Safety and toxicity

Adverse events are summarised in Table 1. The most common AEs related to rIL-21 or rIL-21 plus cetuximab (affecting ⩾20% of subjects), were lethargy, nausea/vomiting, stomatitis, lymphopenia and pyrexia. The majority of AEs were grade 1 (67%) and grade 2 (26%). No DLTs were observed in the 3, 10 or 30 *μ*g kg^−1^ cohorts. In the 100 *μ*g kg^−1^ cohort one patient experienced grade 3 diarrhoea. This was evaluated as possibly related to rIL-21 and probably related to cetuximab. The cohort was subsequently expanded to include six patients. There were no further DLTs at the 100 *μ*g kg^−1^ dose level. No grade 4 toxicity was observed. Rash, a known side effect of cetuximab was observed in eight subjects (53%) but rIL-21 was not considered to have exacerbated skin toxicity in these patients. The trial was prematurely terminated and no subjects were recruited in the 200 and 300 *μ*g kg^−1^ rIL-21 dose groups due to the sponsor's commercial decision to out-license rIL-21. Hence, the MTD was not determined in the present study. Further investigations of rIL-21 in combination with cetuximab at dose levels of 200 *μ*g kg^−1^ and 300 *μ*g kg^−1^ would be required to determine MTD for this treatment regimen.

### Pharmacokinetics

Pharmacokinetic data for rIL21 and cetuximab after single (visit 3, day 8) and multiple (visit 8, day 43) intravenous doses are shown in Tables 2 and 3, respectively. Most serum rIL-21 levels obtained in the 3 *μ*g kg^−1^ and 10 *μ*g kg^−1^ were below the lower limit of quantification (LLOQ) following single and multiple rIL-21 dosing, therefore PK parameters were not calculated for these groups. There was no alteration in the PK of cetuximab when administered in combination with rIL-21.

### Pharmacodynamics

#### Biomarkers

Redistributions of NK cells, cytotoxic T cells, and B cells, as assessed by statistically significant acute drops in absolute numbers of NK cells (CD45+/CD3-/CD16+/CD56+, 87.7 *vs* 42.0 cells *μ*l^−1^, *P*<0.0001), cytotoxic T cells (CD45+/CD3+/CD8+, 217 *vs* 119 cells *μ*l^−1^, *P*<0.0001) and B cells (CD45+/CD19+, 131 *vs* 92 cells *μ*l^−1^, *P*=0.0297) were observed at 24 h post dosing. A clear drop in T-helper cells (CD45+/CD3+/CD4+, 403 *vs* 310 cells *μ*l^−1^, *P*=0.0876) were also observed, but this did not reach statistical significance. A statistically significant increase at 24 h post dosing in the absolute number of monocytes (CD45+/CD14+/CD64+, 249 *vs* 396 cells *μ*l^−1^, *P*=0.0005) was found. In addition, a significant increase in expression (MESF) of the ADCC mediating Fc*γ*RI (CD64) on monocytes was observed at 24 h post dosing (111.7 *vs* 156.9 molecules of equivalent soluble fluorochrome, *P*<0.0001). No statistically significant effects of rIL-21 dosing on NK cytotoxicity or ADCC activity was detected. However, in a total of 9 out of 14 patients in which serial samples were evaluable for NK cytotoxicity towards K562 cells at an effector to target ratio of 50 : 1, increased levels of NK cytotoxicity were observed at 24 h and 1 week post dosing (mean NK kill 13.6% (±s.e.m. 4.14) on day 8, pre-dose; 16.6% (±s.e.m. 4.49) at 24 h post dose and 19.8% (±s.e.m. 4.41) at day 15, one week post dose (*n*=14 patients).

**Figure 3:**
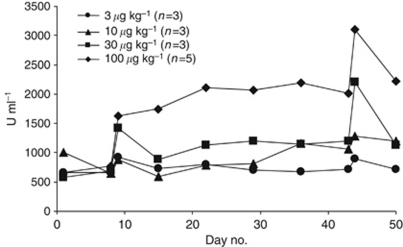
Mean serum sCD25 (U ml^−1^).

Soluble IL-2 receptor (sCD25) is cleaved from T and NK cells on activation and was measured as a marker of immune activation following rIL-21 administration. A statistically significant dose-dependent increase in serum levels of sCD25 (Figure 3) was observed (*P*<0.0001).

#### Immunogenicity

Of the 15 subjects, anti rIL-21 antibodies were detected in only one subject at day 50. Because of insufficient volume, further evaluation of the positive sample for neutralising antibodies was not possible.

### Clinical outcomes

In all, 14 of the 15 patients entered into the study had a tumour assessment performed after 8 weeks of therapy. One patient in the 100 *μ*g kg^−1^ cohort was unable to have a tumour assessment because of treatment-related toxicity (diarrhoea). Of these 15 patients, 8 received treatment in the extension trial and 3 of these had a tumour assessment performed at week 16. Responses are summarised below in Table 4.

## Discussion

This phase 1 trial of rIL-21 administered once weekly in combination with cetuximab to patients with previously untreated stage IV CRC was terminated prematurely because ofthe sponsor's decision to divest the entire programme. Overall, rIL-21 at doses of 3, 10, 30 and 100 *μ*g kg^−1^ administered i.v. once weekly was well tolerated in combination with cetuximab 250 mg m^−2^ once weekly. Adverse events were mild and similar to those previously reported for rIL-21 (Davis *et al*, 2007; Thompson *et al*, 2008). Of note, rash was reported as a common AE in trials of single agent rIL-21. Concerns that the combination of rIL-21 with cetuximab, also documented to cause rash, might lead to enhanced cutaneous toxicity were not borne out in this trial. One of six patients at the expanded 100 *μ*g kg^−1^ dose level experienced a DLT (grade 3 diarrhoea) and no grade 4 toxicity was reported. The maximum administered dose of rIL-21 was 100 *μ*g kg^−1^ for this combination schedule with cetuximab. Further investigations of rIL-21 in combination with cetuximab at dose levels of 200 and 300 *μ*g kg^−1^ would be required to determine the MTD for this treatment regimen.

The increase in AUC observed with escalating doses of rIL-21 following single and multiple dosing with 30 and 100 *μ*g kg^−1^ rIL-21 was linear in nature. As, in the lowest two dose cohorts, rIL-21 levels were below the LLOQ, accurate conclusions cannot be drawn regarding the linearity or otherwise of serum rIL-21 levels following dosing. No interaction between the PK of rIL-21 and cetuximab was identified and the elimination half life of cetuximab was in accordance with that found in previous studies of cetuximab administered as a single agent (70–100 h)(Reynolds and Wagstaff, 2004; Delbaldo *et al*, 2005).

Despite the rapid clearance from the blood of rIL-21, immune activation is clearly demonstrated in this study by a dose-dependent rise in sCD25, indicating soluble IL-2 receptor cleavage. The data showing redistribution of ADCC competent CD16+ NK cells and CD64+ monocytes as well as cytotoxic T cells and B cells is in line with previous observations from clinical trials and provide further evidence of biological activity of this treatment combination (Davis *et al*, 2009). Moreover, the apparent, yet not statistically significant, increase in NK cytotoxicity towards K562 cells in a subset of the patients is also in alignment with previous observations and further supports the immunological rationale for combining rIL-21 with ADCC-mediating compounds such as cetuximab (Frederiksen *et al*, 2008; Thompson *et al*, 2008). Few conclusions can be drawn from the detection of anti-rIL-21 antibodies in one subject as insufficient sample material prevented further analyses.

Nine subjects (60%) had stable disease as their best response. There are no response data available for cetuximab monotherapy in previously untreated patients with advanced CRC, however, the disease control rate for cetuximab alone in patients who had failed prior chemotherapy has been documented as 32.4 (Cunningham *et al*, 2004) and 39.4% (Jonker *et al*, 2007). In the context of a phase 1 study, therefore, the results achieved are very encouraging. The previously untreated patients in the current study might be expected to have a more favourable outcome from cetuximab alone; however, it is possible that rIL-21 has contributed to the disease stabilisation in these patients without significant added toxicity. The role of tumour K-ras and EGFR status was not known at the time of study inception and is a factor, which may have influenced clinical outcomes. Unfortunately, tumour tissue was not collected to allow retrospective analysis. Numbers are insufficient to conclude whether there is a dose-response relationship for efficacy but the proportion of patients with stable disease in the 100 *μ*g kg^−1^ cohort without undue toxicity suggests that this may be a biologically effective dose.

This study has confirmed the feasibility of combining rIL-21 with cetuximab, both administered on a weekly schedule, in patients with stage IV CRC. Further studies using this combination would be required to determine the MTD and potential efficacy of this promising regimen.

## Figures and Tables

**Figure 1:**
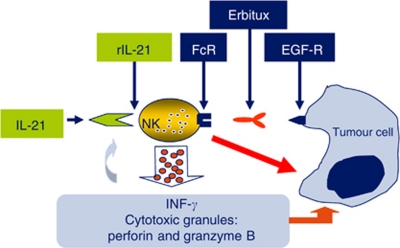
Proposed mechanism for enhanced anti-tumour activity when combining rIL-21 and cetuximab.

**Table 1:** Summary of adverse events related to either rIL-21 or rIL-21 plus cetuximab

	**rIL-21 dose level**				
**Adverse event**	**3 (*n*=3)**	**10 (*n*=3)**	**30 (*n*=3)**	**100 (*n*=6)**	**Total**
*Clinical*					
Lethargy	2	3	2	6 (1)	(1)
Diarrhoea				2 (1)	(1)
Nausea				4	4
Vomiting				3	3
Stomatitis		3		1	4
Dyspepsia			1	1	2
Abdo pain				1	1
Chelitis	1				1
Constipation			1		1
Dry mouth		1			1
Mouth ulcers				1	1
Palpitations	1				1
Tachycardia			1		1
Eye pain	1		1		2
Dry eyes		1	1		2
Conjunctivitis	1				1
Pyrexia			1	2	3
Chills				1	1
Anorexia				1	1
Nail bed infection	1				1
Arthralgia				1	1
Nasal mucosal disorder	1	1			2
Epistaxis	1	1			2
Cough		1			1
Alopecia				1	1
Dry skin		1			1
Erythema		1			1
Palmar plantar erythema				1	1
Rash	2				2
Skin exfoliation			1	1	2
					
*Laboratory*					
Lymphopenia			1 (1)	2 (2)	3 (3)
Hypocalcaemia				1	

Number of patients with a grade 3 adverse event is shown in parenthesis.

**Table 2:** Summary of pharmacokinetic endpoints for rIL-21 following single (day 8) and multiple (day 43) doses (once weekly) of intravenous rIL-21 in combination with once weekly dosing of cetuximab

	**Day 8 rIL-21 dose (*μ*g kg^−1^)**				**Day 43 rIL-21 dose (*μ*g kg^−1^)**			
Parameter	3	10	30	100	3	10	30	100
AUC_0−24 h_ (*μ*g h l^−1^)	—	—	85	617	—	—	115	487
AUC_0−168h_ (*μ*g h l^−1^)	—	—	85	623	—	—	115	489
AUC_0−INF_ (*μ*g h l^−1^)	—	—	85	623			115	489
Concentration (*C*_5 min_) post i.v. (*μ*g l^−1^)	—	18	75	435	4.8	3.2	74	286
*t*_½_ (h)	—	—	2.1	3.6	—	—	2.1	2.7
CL (ml h^−1^ kg^−1^)	—	—	350	160	—	—	260	204
Vz (ml kg^−1^)	—	—	1080	829	—	—	699	607
MRT (h)	—	—	2.3	3.5	—	—	2.7	3.1

Abbreviations: CL=clearance; MRT=mean residence time; Vz=terminal Vd.

**Table 3:** Summary of pharmacokinetic endpoints for cetuximab following single (day 8) and multiple (day 43) doses (once weekly) of cetuximab in combination with once weekly dosing of rIL-21

	**Day 8 cetuximab dose rIL-21 (*μ*g kg^−1^)**				**Day 43 cetuximab dose rIL-21 (*μ*g kg^−1^)**			
Parameter	3	10	30	100	3	10	30	100
AUC_0−168 h_ (*μ*g h l^−1^)	14 798	11 732	11 268	15 776	10 859	16 576	11 971	12 660
AUC_0−INF_ (*μ*g h l^−1^)	18 608	14 489	12 850	21 175	13 599	25 699	14 522	15 817
Concentration (*C*_5 min_) i.v. (*μ*g l^−1^)	183	139	160	179	149	173	164	81
*t*_½_ (h)	74	59	54	88	72	112	67	73
CL (ml h^−1^ kg^−1^)	17	21	22	16	23	15	21	19.7
Vz (ml kg^−1^)	1432	1675	1443	1679	2126	2202	1614	1680
MRT (h)	89	88.0	67	108	94	151	80	89

Abbreviations: CL=clearance; MRT=mean residence time; Vz=terminal Vd.

**Table 4:** Summary of clinical responses to rIL-21 and cetuximab

		**Response week 8 (*n*=14)**			**Response week 16 (*n*=3)**		**Best overall response (*n*=15)**		
**Dose level (*μ*g kg^−1^)**	**Total patients**	**SD**	**PD**	**NE**	**SD**	**PD**	**SD**	**PD**	**NE**
3	3	1	2	0	NA	NA	1	2	0
10	3	3	0	0	0	2	3	0	0
30	3	1	2	0	NA	NA	1	2	0
100	6	4	1	1	1	0	4	1	1

Abbreviations: NA=not applicable; NE=non-evaluable; PD=progressive disease; SD=stable disease.

## References

[bib1] Ackland SP, Jones M, Tu D, Simes J, Yuen J, Sargeant AM, Dhillon H, Goldberg RM, Abdi E, Shepherd L, Moore MJ (2005) A meta-analysis of two randomised trials of early chemotherapy in asymptomatic metastatic colorectal cancer. Br J Cancer 93: 1236–1243 16265352 10.1038/sj.bjc.6602841 PMC2361520

[bib2] Bokemeyer C, Bondarenko I, Makhson A, Hartmann JT, Aparicio J, de Braud F, Donea S, Ludwig H, Schuch G, Stroh C, Loos AH, Zoubel A, Koralewski P (2009) Fluorouracil, leucovorin, and oxaliplatin with and without cetuximab in the first-line treatment of metastatic colorectal cancer. J Clin Oncol 27: 663–671 19114683 10.1200/JCO.2008.20.8397

[bib3] Cunningham D, Humblet Y, Siena S, Khayat D, Bleiberg H, Santoro A, Bets D, Mueser M, Harstrick A, Verslype C, Chau I, Van Cutsem E (2004) Cetuximab monotherapy and cetuximab plus irinotecan in irinotecan-refractory metastatic colorectal cancer. N Engl J Med 351: 337–345 15269313 10.1056/NEJMoa033025

[bib4] Davis ID, Brady B, Kefford RF, Millwrd M, Cebon J, Skrumsager BK, Mouritzen U, Hansen LT, Skak K, Lunsgaard D, Frederiksen KS, Kristjansen PE, McArthur G (2009) Clinical and biological efficacy of recombinant human interleukin-21 in patients with stage IV malignant melanoma without prior treatment: a phase IIa trial. Clin Cancer Res 15: 2123–2129 19276257 10.1158/1078-0432.CCR-08-2663

[bib5] Davis ID, Skrumsager BK, Cebon J, Nicholaou T, Barlow JW, Moller NP, Skak KL, Lundsgaard D, Frederiksen KS, Thygesen P, McArthur GA (2007) An open-label, two-arm, phase I trial of recombinant human interleukin-21 in patients with metastatic melanoma. Clin Cancer Res 13: 3630–3636 17575227 10.1158/1078-0432.CCR-07-0410

[bib6] Delbaldo C, Pierga JY, Dieras V, Faivre S, Laurence V, Vedovato JC, Bonnay M, Mueser M, Nolting A, Kover A, Raymond E (2005) Pharmacokinetic profile of cetuximab (erbitux) alone and in combination with irinotecan in patients with advanced EGFR-positive adenocarcinoma. Eur J Cancer 41: 1739–1745 16051481 10.1016/j.ejca.2005.04.029

[bib7] Frederiksen KS, Lundsgaard D, Freeman JA, Hughes SD, Holm TL, Skrumsager BK, Petri A, Hansen LT, McArthur GA, Davis ID, Skak K (2008) IL-21 induces *in vivo* immune activation of NK cells and CD8(+) T cells in patients with metastatic melanoma and renal cell carcinoma. Cancer Immunol Immunother 57: 1439–1449 18286285 10.1007/s00262-008-0479-4 PMC2491425

[bib8] Jonker DJ, O'Callaghan CJ, Karapetis CS, Zalcberg JR, Tu D, Au Hj, Berry SR, Krahn M, Price T, Simes RJ, Tebbutt NC, van Hazel G, Wierzbicki R, Langer C, Moore MJ (2007) Cetuximab for the treatment of colorectal cancer. N Engl J Med 357: 2040–2048 18003960 10.1056/NEJMoa071834

[bib9] Reynolds NA, Wagstaff AJ (2004) Cetuximab in the treatment of metastatic colorectal cancer. Drugs 64: 109–118; discussion 119-121 14723561 10.2165/00003495-200464010-00007

[bib10] Roda JM, Joshi T, Butchar JP, McAlees JW, Lehman A, Tridandapani S, Carson III WE (2007) The activation of natural killer cell effector functions by cetuximab-coated, epidermal growth factor receptor positive tumor cells is enhanced by cytokines. Clin Cancer Res 13: 6419–6428 17962339 10.1158/1078-0432.CCR-07-0865

[bib11] Thompson JA, Curti BD, Redman BG, Bhatia S, Weber JS, Agarwala SS, Sievers EL, Hughes SD, DeVries TA, Hausman DF (2008) Phase I study of recombinant interleukin-21 in patients with metastatic melanoma and renal cell carcinoma. J Clin Oncol 26: 2034–2039 18347008 10.1200/JCO.2007.14.5193

[bib12] Van Cutsem E, Kohne CH, Hitre E, Zaluski J, Chang Chien CR, Makhson A, D'Haens G, Pinter T, Lim R, Bodoky G, Roh JK, Folprecht G, Ruff P, Stroh C, Tejpar S, Schlichting M, Nippgen J, Rougier P (2009) Cetuximab and chemotherapy as initial treatment for metastatic colorectal cancer. N Engl J Med 360: 1408–1417 19339720 10.1056/NEJMoa0805019

[bib13] Watanabe M, Kono K, Kawaguchi Y, Mizukami Y, Mimura K, Maruyama T, Fujii H (2010) Interleukin-21 can efficiently restore impaired antibody-dependent cell-mediated cytotoxicity in patients with oesophageal squamous cell carcinoma. Br J Cancer 102: 520–529 20029417 10.1038/sj.bjc.6605502 PMC2822939

